# Exploring the cost implications of different treatment modalities for uterine fibroids under the DRG system

**DOI:** 10.3389/fpubh.2025.1660033

**Published:** 2025-11-25

**Authors:** Kun Tan, Linya Huang, Yuxian Nie, Qiuling Shi, Qiushuo Liu, Su Yan, Ting Lu, Xite Yang, Yongzeng Lai, Xiaofeng Zhu

**Affiliations:** 1 Sichuan Provincial Health Information Center, Chengdu, Sichuan, China; 2 State Key Laboratory of Ultrasound in Medicine and Engineering, College of Biomedical Engineering, Chongqing Medical University, Chongqing, China; 3 Medical Records Management Center, Suining Central Hospital, Suining, Sichuan, China; 4 Business School, Sichuan University, Chengdu, China; 5 Department of Mathematics, Wilfrid Laurier University, Waterloo, ON, Canada

**Keywords:** uterine fibroids, high-intensity focused ultrasound, diagnosis-related group system, medical resource consumption, cost drivers

## Abstract

This paper, based on data from inpatient medical records with uterine fibroids (ICD-10: D25) from the medical record homepages of secondary and higher-level hospitals in Sichuan Province between 2016 and 2024, investigated differences in medical resource consumption and costs between high-intensity focused ultrasound (HIFU) and traditional surgical treatments under the diagnosis-related group (DRG) system. Cases were classified using the MS-DRG grouper into groups with and without complications or comorbidities (CC/MCC). An XGBoost model was employed to reclassify data for HIFU patients, addressing missing coding. Group validity was assessed using the coefficient of variation (CV) and reduction in variance (RIV). Factors influencing costs were identified via multifactorial regression analysis. Results showed that in the group without CC/MCC, HIFU treatment significantly reduced the length of hospital stay, decreased the proportion of consumables costs and medication costs, but increased the proportion of treatment costs. Median hospitalization costs were significantly higher in the CC/MCC group than in the non-CC/MCC group. Multifactorial regression analysis identified length of stay (LOS), HIFU treatment, and CC/MCC grouping as key cost drivers. Additionally, costs for patients covered by Urban Employee Basic Medical Insurance and Commercial Health Insurance were significantly higher than those with other payment types. This paper confirms the effectiveness of DRG grouping in reflecting resource consumption disparities and reveals the potential of HIFU technology for optimizing medical resource allocation. Recommendations include promoting HIFU adoption, optimizing medical insurance payment policies, and strengthening hospital management to achieve dual goals of cost control and healthcare quality improvement. The findings provide empirical evidence for DRG payment reform and the selection of uterine fibroid treatment modalities.

## 1 Introduction

Uterine leiomyoma (UL), the most common benign tumor of the female reproductive system, predominantly affects women aged 30–50 years, with those aged 40–50 accounting for 51.2%−60.9% of cases (1–3). Pathologically, UL is characterized by the abnormal proliferation of smooth muscle cells in the myometrium and the deposition of an extracellular matrix rich in collagen, fibronectin, and proteoglycans. The clinical manifestations exhibit significant heterogeneity: approximately 30% of patients present with classic symptoms such as menorrhagia, pelvic pressure, and anemia (4–7), while UL is also closely associated with reproductive dysfunction, including infertility, recurrent miscarriage, and preterm delivery (8, 9). Notably, about 70% of uterine sarcoma cases have a history of UL (10), highlighting its role as a critical target in gynecologic oncology prevention.

Diagnosis follows a four-tiered evaluation system (6): pelvic examination (specificity: 82%); Transvaginal ultrasound (sensitivity: 91% when uterine volume < 375 ml and ≤ 4 fibroids); Saline infusion sonohysterography; MRI as the gold standard (localization accuracy: 98%), essential for differentiating UL from uterine sarcoma (3% of uterine tumors) (11), Sarcomas are distinguished histologically by mitotic index (>10/10 high-power fields), cellular atypia, and coagulative necrosis (12). UL significantly contributes to global rates of hysterectomy. In the United States, approximately 600,000 annual procedures result in direct medical costs of $2.2 billion (13, 14). In China, the incidence of UL rose from 3.4% in the 1970s to 5.2% in 2012, with over 100,000 surgeries performed annually, imposing substantial socioeconomic burdens. Optimizing treatment strategies and cost-control measures is thus imperative (15).

Diagnosis-related groups (DRG), an advanced payment management model developed by Yale University and first implemented in the US in 1983 (16), classifies patients based on clinical similarity, with resource consumption homogeneity as a secondary principle (17, 18). It establishes standardized reimbursement through parameters including primary diagnosis, procedure type, age, length of stay (LOS), and complications. Studies confirm DRG's efficacy in controlling costs, reducing patient financial burdens, and ensuring quality by denying payment for unnecessary or excessive services (19). International DRG practices vary markedly: Ireland classifies appendectomies into two groups by surgical complexity and age, while Germany subdivides them into 11 groups; France employs multivariate parameters (age, comorbidities, and LOS), whereas Austria considers only age (20, 21). Since China's DRG reform was launched in 200 (22), multiple standards (BJ-DRG, CN-DRG, etc.) have emerged. In 2019, the National Healthcare Security Administration unified pilot cities under CHS-DRG, though regional economic disparities permit localized adaptations, resulting in significant policy heterogeneity (23). As a tool for balancing stakeholder interests and optimizing care delivery, DRG has become central to global healthcare resource allocation and payment reforms (24).

Research gaps in DRG for UL treatment include: (1) limited scope. Most studies focus on traditional surgeries (hysterectomy/hysteroscopy), neglecting minimally invasive alternatives like high-intensity focused ultrasound (HIFU) (25, 26), creating blind spots in resource-consumption analysis. (2) Methodological constraints: conventional techniques (logistic regression/E-CHAID) fail to capture non-linear complexities, thereby limiting the precision of grouping. (3) Generalizability issues. Single-center studies with small samples lack regional representativeness (23, 27, 28).

This paper utilizes large-scale medical record data (2016–2024) from hospitals in Sichuan Province to compare HIFU with traditional surgical methods. We employ MS-DRG grouping augmented by XGBoost machine learning to address HIFU coding gaps, ensuring analytical rigor. Multifactorial regression identifies key cost drivers (treatment modality, complexity, and insurance type).

Results demonstrate HIFU's advantages: reduced LOS, lower consumable/drug costs (offset by higher procedure fees), and long-term efficiency. By bridging the research gaps in DRG for UL therapies, this work provides evidence for policymaking and hospital management.

## 2 Materials and data pre-processing

This paper selects the relevant information from the first page of medical records of patients diagnosed with uterine fibroids (ICD-10 diagnostic code D25) in secondary and tertiary institutions in Sichuan Province from 2016 to 2024. During the initial data cleaning phase, a total of 11,844 cases were excluded due to abnormal hospitalization expenses (i.e., costs outside $\bar{x}\pm 2s$) or those with hospitalization days exceeding 180 days, resulting in 269,755 initial samples. These cases were preliminarily grouped using the MS-DRG grouper and subsequently categorized by treatment method into high-intensity focused ultrasound (HIFU) and traditional surgery groups.

Subsequent analysis identified a systematic misclassification issue affecting HIFU cases. HIFU currently lacks a corresponding code in the MS-DRG system and is not classified as a surgical procedure. As a result, some HIFU patients were incorrectly grouped into categories based on medical management. To ensure grouping accuracy for subsequent comparative cost analysis, data regrouping was performed on the 14,411 HIFU patients from the initial grouping. This subset was verified to have no missing or duplicate values. Using an XGBoost model, 39 cases were re-assigned to the “Non-malignant Uterine and Adnexal Surgery with CC/MCC” group.

In comparison, 14,372 cases were assigned to the “Non-malignant Uterine and Adnexal Surgery without CC/MCC” group. The XGBoost model achieves the best performance (accuracy 0.90, precision 0.88, recall 0.90, and F1 score 0.86) and is employed to correct erroneous groupings caused by missing codes. The final analytical dataset comprised 268,709 cases, including 243,910 patients in the “Non-malignant without CC/MCC” group and 24,799 in the “Non-malignant with CC/MCC” group. This curated dataset provides a reliable foundation for subsequent analysis of cost structure differences across various treatment modalities.

As shown in Table 1, to verify the validity of the grouping procedure, we calculate the coefficient of variation (CV ≤ 0.29) within each subgroup and the reduction in variance (RIV = 78.27%) between two subgroups. The CV values for both the Without CC/MCC group and the With CC/MCC (Complication/Comorbidity and Major Complication/Comorbidity) group are ≤ 0.8, indicating slight within-group variation and good within-group homogeneity for each group. The RIV is 78.27, indicating good between-group heterogeneity. This lends support to the machine learning method used to classify patients with missing codes.

**Table 1:** CV and RIV values for total hospitalization costs of uterine fibroid patients in different DRG subgroups.

**Within-group CV values**		**Between-group RIV value (%)**
**Without CC/MCC**	**With CC/MCC**	
0.28	0.29	78.27

## 3 Statistical comparisons of costs between patient groups

### 3.1 Costs between DRG groups

To identify patterns of costs and resource utilization for different DRG groups, we calculate the 25%, 50% (median), and 75% percentiles of the length of stay, total costs, and cost components for Without CC/MCC and With CC/MCC cases, respectively. The non-parametric Kruskal–Wallis *H* tests are conducted to identify whether the distributions of the two groups differ significantly from each other. Table 2 reports the results. From the descriptive statistics for each group, the central locations of total costs vary, with the CC/MCC group exhibiting a higher cost at all percentiles considered. According to the Kruskal–Wallis *H* test, different DRG subgroups show significant differences in length of stay (*H* = 8,162.00, *P* < 0.001), total cost (*H* = 3,101.37, *P* < 0.001), medical service cost (*H* = 857.23, *P* < 0.001), diagnostic cost (*H* = 19.70, *P* < 0.001), treatment cost (*H* = 1,891.17, *P* < 0.001), medication cost (*H* = 1,988.26, *P* < 0.001), and consumables cost (*H* = 61.84, *P* < 0.001).

**Table 2:** Hospitalization costs and composition of uterine fibroid patients in different DRG subgroups.

**Statistical measure**	**Length of stay (d)**	**Hospitalization cost (¥)**	**Medical service cost (%)**	**Diagnostic cost (%)**	**Treatment cost (%)**	**Medication cost (%)**	**Consumables cost (%)**
**Without CC/MCC group (*****N*** = **243,910)**							
Median	7	12,476.20	8.75%	21.16%	37.51%	12.30%	10.50%
25%	5	9,256.50	5.86%	16.65%	29.72%	8.77%	5.89%
75%	9	15,909.71	12.86%	26.06%	45.52%	16.99%	16.95%
**With CC/MCC group (*****N*** = **24,799)**							
Median	9	14,451.68	9.84%	21.48%	33.91%	14.14%	10.89%
25%	7	10,686.96	6.80%	16.59%	26.85%	10.47%	6.60%
75%	12	18,616.89	14.01%	26.62%	41.60%	19.12%	16.65%
Kruskal–Wallis *H* Test	8,162.00	3,101.37	857.23	19.70	1,891.17	1,988.26	61.84
*P*-value	0.00	0.00	0.00	0.00	0.00	0.00	0.00

¥1 ≈ USD 0.14, based on the 2024 average exchange rate.

Specifically, regarding the length of stay, the median length of stay in the With CC/MCC group was 9 days, significantly longer than the 7 days in the Without CC/MCC group (difference, 2 days; *P* < 0.001). Regarding total cost, the median cost in the With CC/MCC group was 14,452 yuan, significantly higher than the 12,476 yuan in the Without CC/MCC group (difference, 1,976 yuan; *P* < 0.001). Furthermore, the medication cost proportion (median 14.14%) and consumables cost proportion (10.89%) in the With CC/MCC group were both higher than in the Without CC/MCC group (medication cost, 12.30%; consumables cost, 10.50%). In comparison, the treatment cost proportion (33.91%) was lower than in the Without CC/MCC group (37.51%), indicating greater treatment resource consumption in the With CC/MCC group (complex condition group).

### 3.2 Costs between HIFU and surgical groups

We then compare the costs and resource utilization between HIFU-treated and surgery-treated patients. As the selection of HIFU and surgery procedure may be systematically affected by other factors and is thus non-random, directly comparing the costs between HIFU and surgical cases may confound the effect caused by the treatment method itself with those caused by other factors. To address this issue, we apply propensity score matching to create two matched samples with similar baseline characteristics but receiving different treatments (HIFU vs. surgery). Propensity scores are calculated using a logistic regression incorporating age, discharge year, length of stay, marital status, insurance type, and CC/MCC status. Matching is then performed using the nearest neighbor scheme, resulting in two matched samples of 19,232 patients each. As shown in Table 3, covariate imbalance was substantial before matching (e.g., SMD for length of stay = −1.43; CC/MCC status = 0.77), but after matching, the majority of standardized mean differences were reduced to below 0.1, indicating good balance. As a result, cost comparisons between matched groups should mainly reflect differences attributable to the choice of treatment rather than underlying patient characteristics.

**Table 3:** Covariate balance results before and after matching.

**Characteristic**	**Before matching**			**After matching**		
	**HIFU (*****n*** = **19,232)**	**Surgery (*****n*** = **249,477)**	**SMD**	**HIFU (*****n*** = **19,232)**	**Surgery (*****n*** = **19,232)**	**SMD**
Age	42.48	45.66	−0.48	42.48	42.65	−0.03
Discharge year (2017=0)	3.13	3.13	0.00	3.13	3.36	−0.12
Length of stay	3.98	8.08	−1.43	3.98	4.13	−0.05
**Marriage status, %**						
Other	5.70%	2.50%	0.14	1.90%	1.70%	0.07
Divorced	1.90%	1.80%	0.00	5.70%	4.20%	0.01
Unmarried	3.80%	2.80%	0.05	3.80%	3.70%	0.01
Married	88.70%	92.90%	−0.13	88.70%	90.40%	−0.06
**Insurance type, %**						
Urban employee basic Med Ins	26.20%	22.90%	0.08	26.20%	28.90%	−0.06
Poverty assistance	0.00%	0.80%	−0.51	0.00%	0.00%	−0.01
Other	0.60%	3.20%	−0.34	0.60%	0.30%	0.04
Other social insurance	2.70%	7.80%	−0.31	2.70%	2.30%	0.03
Fully government-funded	0.10%	0.40%	−0.14	0.10%	0.00%	0.01
Fully self-paid	14.90%	9.60%	0.15	14.90%	18.60%	−0.10
Commercial medical Ins	0.10%	0.50%	−0.11	0.10%	0.10%	0.00
New rural cooperative Med	15.20%	14.00%	0.03	15.20%	10.60%	0.13
Urban resident basic Med Ins	40.20%	40.80%	−0.01	40.20%	39.20%	0.02
Without CC/MCC	98.70%	90.20%	0.77	98.70%	98.80%	−0.01
With CC/MCC	1.30%	9.80%	−0.77	1.30%	1.20%	0.01

**Figure 1:**
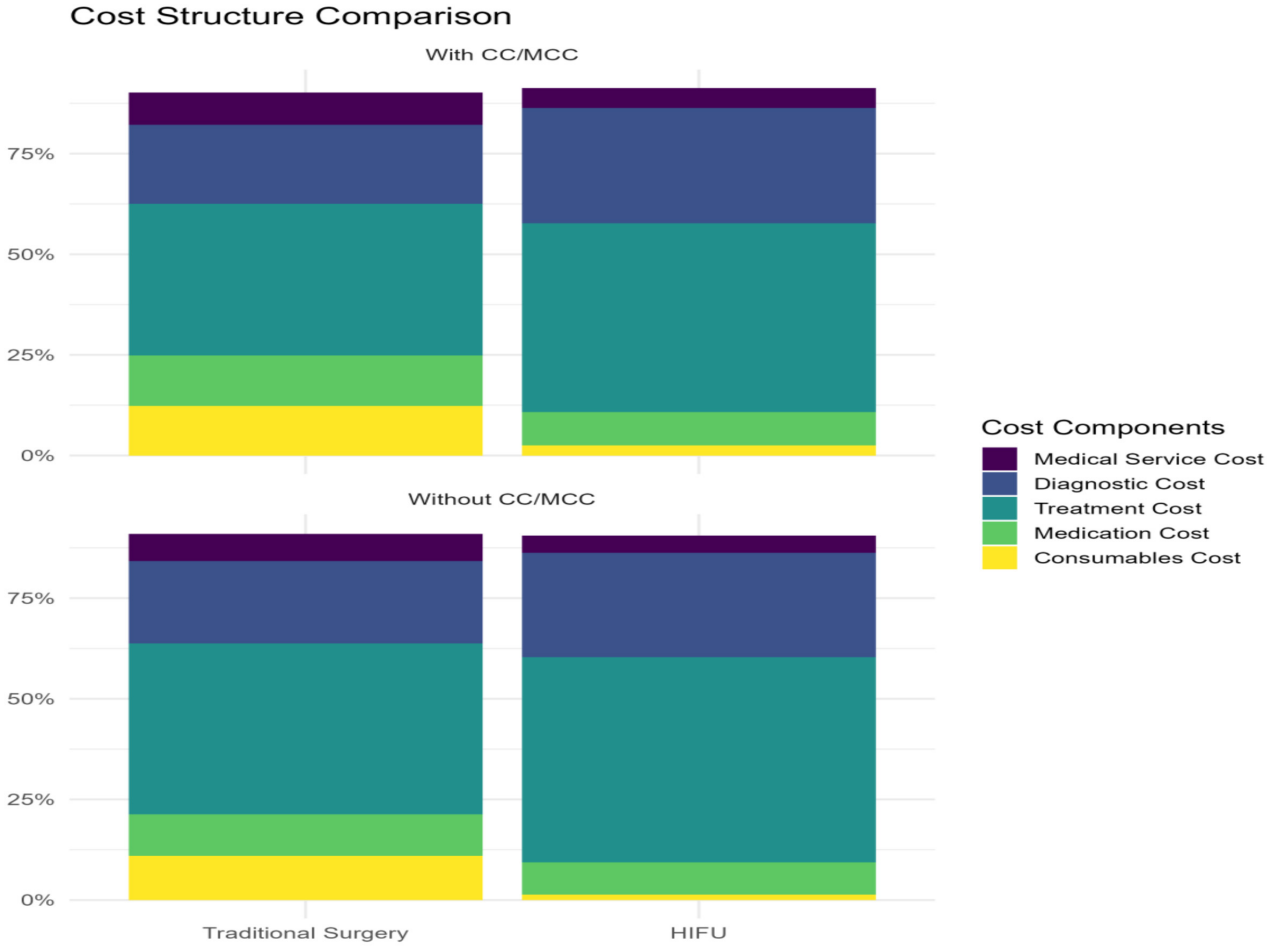
Cost structure comparison between HIFU and surgery group.

After matching, significant differences remained in cost structure and hospitalization outcomes (see Table 4 and Figure 1). In the without CC/MCC group, HIFU patients had shorter median length of stay (3 vs. 4 days, *P* < 0.001), higher treatment cost proportion (50.99 vs. 42.46%, *P* < 0.001), and markedly lower consumables costs (1.35 vs. 10.94%, *P* < 0.001), while overall hospitalization costs were higher in the HIFU group (¥13,590 vs. ¥9,175, *P* < 0.001). In the CC/MCC group, HIFU patients also showed shorter stays (4 vs. 6 days, *P* < 0.001), reduced consumables and medication cost shares, and higher treatment and diagnostic cost proportions compared with surgery patients. These results confirm that HIFU is associated with distinct patterns of resource utilization, manifested by shorter hospitalization and lower reliance on consumables but higher treatment costs than traditional surgery.

**Table 4:** Analysis of medical cost differences between matched HIFU group and surgery group.

**Treatment method**	**Cases**	**Length of stay (d)**	**Hospitalization cost (¥)**	**Medical service cost (%)**	**Diagnostic cost (%)**	**Treatment cost (%)**	**Medication cost (%)**	**Consumables cost (%)**
**Without CC/MCC group (*****N*** = **37,995)**								
HIFU	18,991	3	13,590.36	4.26%	25.94%	50.99%	8.00%	1.35%
Traditional Surgery	19,004	4	9,174.66	6.78%	20.42%	42.46%	10.39%	10.94%
Kruskal–Wallis *H* Test		72.15	9,612.89	3,464.19	3,326.20	993.23	3,626.98	14,066.49
*P*-value		0.00	0.00	0.00	0.00	0.00	0.00	0.00
**With CC/MCC group (*****N*** = **469)**								
HIFU	241	4	14,703.01	4.97%	28.65%	46.84%	8.33%	2.49%
Traditional Surgery	228	6	13,810.41	9.87%	21.43%	33.84%	14.20%	10.97%
Kruskal–Wallis *H* Test		8.33	13.50	13.27	68.08	17.74	72.65	152.23
*P*-value		0.00	0.00	0.00	0.00	0.00	0.00	0.00

## 4 Multivariate regression analysis for hospitalization costs

### 4.1 Regression on full sample

We use a multivariate regression analysis to examine the statistical significance and marginal effects of different factors that potentially affect the hospitalization costs for patients in our sample. This approach allows for the confounding effects to be further controlled and disentangled by simultaneously including different factors that are associated with the variation in hospitalization costs.

The full-sample regression includes seven independent variables, namely age, discharge year, length of stay, marital status, medical payment method, treatment method, and DRG groups. These variables have been shown to exhibit a significant relationship with the hospitalization cost using a univariate regression analysis (*P* < 0.001). Among these variables, age, discharge year, and length of stay are continuous variables measured in quantities. At the same time, marital status, medical payment method, treatment method, and DRG group are categorical variables that are entered into the regression using one-hot encoding. The reference (or baseline) group is selected to represent a patient who is divorced, covered by urban resident basic medical insurance, and treated with traditional surgery. It belongs to group 3 (without CC/MCC). The regression coefficient associated with a category thus quantifies the expected difference in hospitalization costs between a patient in this category and in the reference category, holding everything else constant.

Before conducting multivariate regression, we assess multicollinearity between independent variables using the generalized variance inflation factor (GVIF), adjusted for degrees of freedom to account for categorical variables. As shown in Table 5, all $GVIF^{\frac{1}{\left(2^{*}Df\right)}}$ values were close to 1 (ranging from 1.01 to 1.13), indicating that multicollinearity is unlikely to be a serious concern in the model, and the unique effect of each variable can be estimated with reasonably high statistical accuracy.

**Table 5:** Generalized variance inflation factor (GVIF).

**Variable**	**GVIF**	**Df**	$\frac{1}{\left(2^{*}\text{Df}\right)}$
Age	1.12	1	1.06
Discharge year	1.17	1	1.08
Length of stay	1.28	1	1.13
Marital status	1.09	4	1.01
Medical payment method	1.25	8	1.01
HIFU	1.11	1	1.05
Group (with or without CC/MCC)	1.04	1	1.02

**Figure 2:**
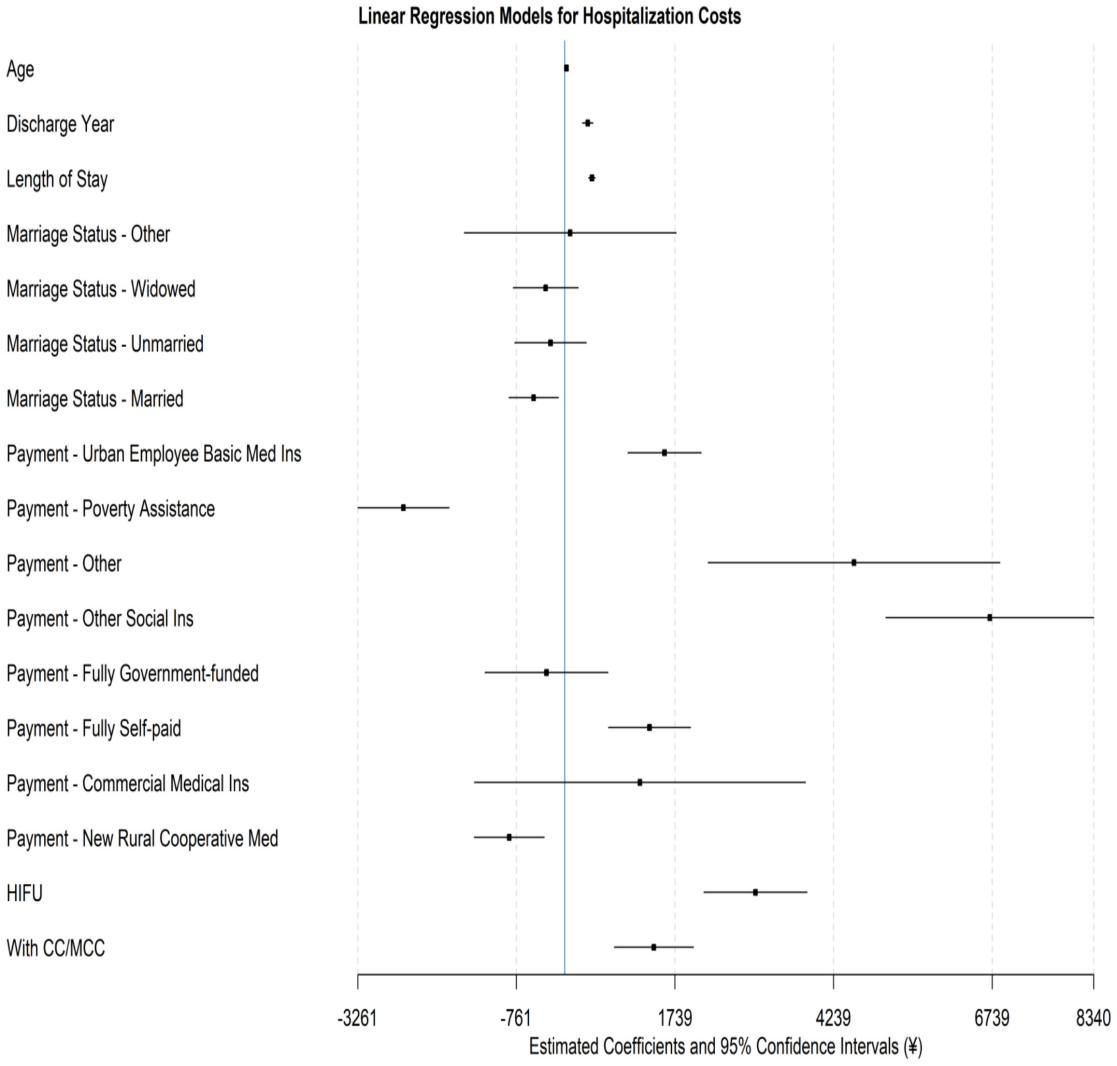
Forest plot for the regression model on hospitalization costs.

As shown in Table 6, multivariate regression results confirm that the majority of variables exhibit significant relationships with hospitalization costs. Figure 2 visualizes the point estimate and confidence intervals of each variable using a forest plot. To further facilitate interpretation, marginal effects were expressed as a percentage of the cost for the reference group (¥6,448 as measured by the intercept term; see Table 6). Among demographic factors, age is shown to exhibit a relatively modest effect: for each 1-year increase in age, the cost increases by 0.44%. While this difference is statistically significant, it may have a limited economic impact in practice, especially when contrasted with the much stronger effects of treatment modality and insurance type. Marital status also induces differences in costs, with married patients having 7.6% lower costs than divorced patients. This could reflect differences in family support, healthcare-seeking behavior, or financial capacity, though the absolute effect size is smaller compared to other predictors.

**Table 6:** Multivariate regression analysis of hospitalization costs.

**Explanatory variable**	**Est. (¥)**	**S.E**.	***P-*value**	**95% CI**	**Effect vs. baseline**
Baseline: Divorced + Urban resident basic Med Ins + Traditional surgery + Group 3	6,447.70	416.76	< 0.001	[5,630.86, 7,264.55]	
Age	28.13	6.58	< 0.001	[15.24, 41.02]	+0.44%
Discharge year	363.46	42.87	< 0.001	[279.43, 447.49]	+5.64%
Length of stay	429.88	26.25	< 0.001	[378.44, 481.32]	+6.67%
**Marital status**					
Other	86.31	852.38	0.919	[−1,584.34, 1,756.96]	+1.34%
Widowed	−300.26	262.18	0.252	[−814.11, 213.60]	−4.66%
Unmarried	−223.36	288.24	0.438	[−788.31, 341.59]	−3.46%
Married	−489.43	198.35	0.014	[−878.18, −100.67]	−7.59%
**Medical payment method**					
Urban employee basic med Ins	1,573.69	295.48	< 0.001	[994.56, 2,152.83]	+24.41%
Poverty assistance	−2,541.92	366.61	< 0.001	[−3,260.47, −1,823.37]	−39.42%
Other	4,560.40	1,173.47	< 0.001	[2,260.42, 6,860.37]	+70.73%
Other social insurance	6,700.51	836.46	< 0.001	[5,061.07, 8,339.96]	+103.92%
Fully government-funded	−287.27	494.45	0.561	[−1,256.37, 681.84]	−4.46%
Fully self-paid	1,337.63	330.09	< 0.001	[690.65, 1,984.60]	+20.75%
Commercial medical Ins	1,186.01	1,331.38	0.373	[−1,423.46, 3,795.49]	+18.39%
New rural cooperative med	−873.76	281.87	0.002	[−1,426.22, −321.29]	−13.55%
HIFU	3,006.73	414.73	< 0.001	[2,193.87, 3,819.60]	+46.63%
Group 4 (With CC/MCC)	1,404.48	318.47	< 0.001	[780.30, 2,028.67]	+21.78%
*F*-test	4,969.84		< 0.001		
*R*^2^ Adj.	0.239				

Length of stay is shown to be a significant determinant of hospitalization costs. Each additional day of hospitalization increased costs by 6.67%, representing a substantial and policy-relevant effect. Given that mean lengths of stay differed substantially between HIFU and surgery, this factor highlights the importance of hospital efficiency initiatives and post-treatment care pathways in containing costs under the DRG system.

Insurance types emerge as one of the strongest drivers of the observed variation in hospitalization costs. Patients covered by Other Social Insurance incur costs 103.9% higher than the baseline, while those with Urban Employee Basic Medical Insurance experience a 24.4% increase in costs. Conversely, Poverty Assistance and New Rural Cooperative Medical patients witness 39.4 and 13.6% lower costs, respectively. These wide disparities underscore the role of insurance coverage design in shaping cost patterns, reflecting differences in reimbursement rates, access to services, and resource utilization. The magnitude of these effects suggests that payment reform and benefit equalization may be critical levers for controlling systemic cost variation.

Treatment modality also exhibits a notable impact. HIFU patients incurred 46.6% higher costs than surgery patients, likely reflecting the higher technical fees associated with this novel technology. However, as shown in earlier analyses of cost structure, these higher treatment fees are partially offset by markedly lower consumables and medication costs, as well as shorter hospital stays. From a policy perspective, while the higher upfront costs of HIFU may present challenges under DRG-based payment, its efficiency advantages suggest potential long-term savings if bundled payments and cost-sharing arrangements account for resource substitution. Furthermore, clinical complexity remained a vital driver: patients with CC/MCC experience 21.8% higher costs than those without CC/MCC, reinforcing the appropriateness of DRG grouping in capturing severity-related cost variation.

### 4.2 Regression for different DRG groups

**Figure 3:**
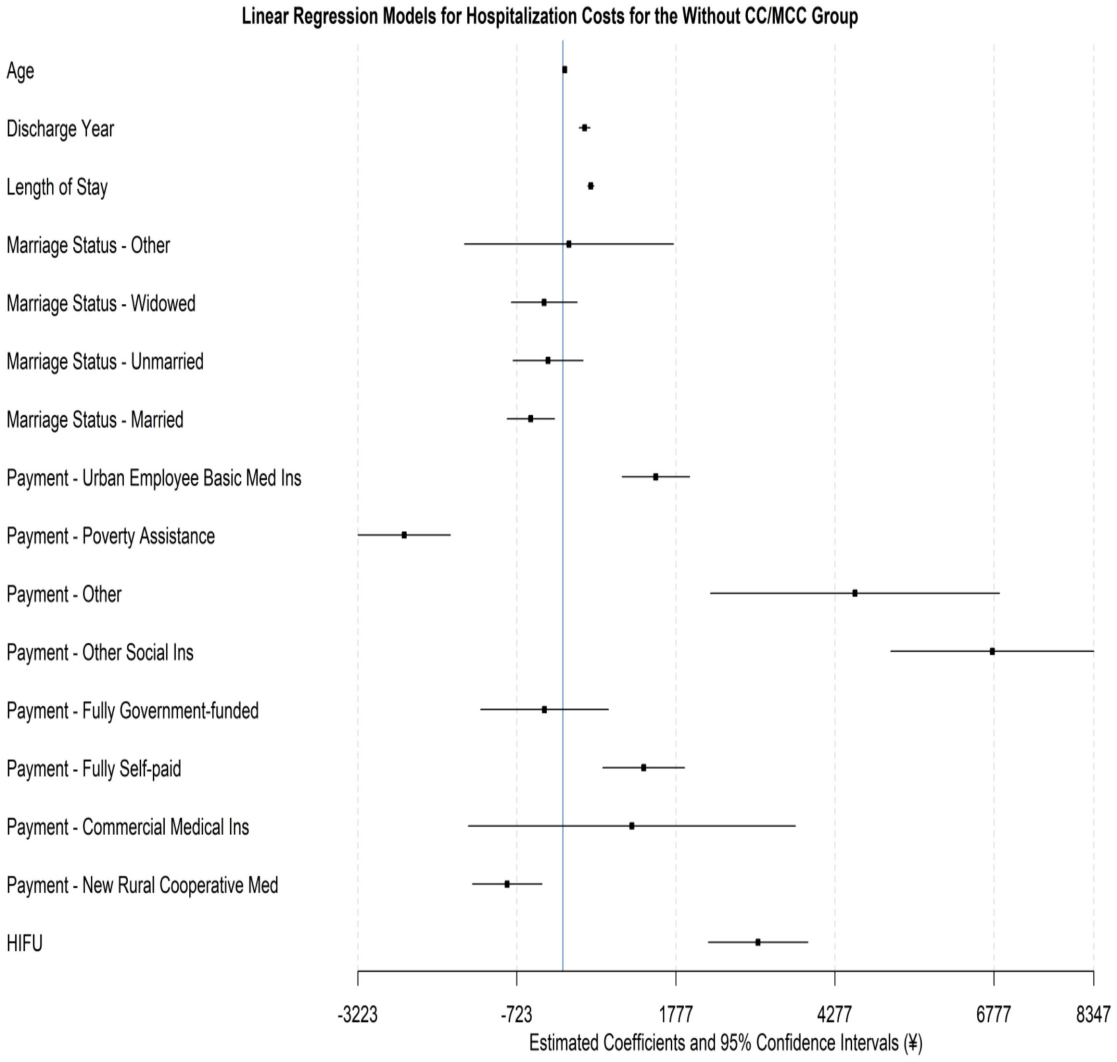
Forest plot for the regression model on hospitalization costs for the without CC/MCC group.

We then estimate the multivariate regression separately for each DRG group, investigating the effects of demographic characteristics, medical payment method, and treatment modality on hospitalization costs for patients with different clinical complexity. The reference group is selected similarly to before, within each DRG group, representing a patient who is divorced, covered by urban resident basic medical insurance, and treated with traditional surgery. Table 7 and Figure 3 show regression results for the Without CC/MCC group. The results suggest that the impact of different factors on hospitalization costs remains significant when only considering patients with less clinical complexity. For each 1-year increase in age, costs increase by 28.48 yuan (+0.44%, *P* < 0.001); for each 1-year increase in the discharge year, costs on average increase by 342.04 yuan (+5.28%, *P* < 0.001). More importantly, an extra day of hospitalization raises costs by 438.00 yuan (+6.76%, *P* < 0.001), again indicating the central role of length of stay in driving expenditures. Regarding marital status, married patients experience costs that are 505.30 yuan lower than those for divorced patients (−7.80%, *P* = 0.008). No significant cost differences are found for unmarried or widowed patients relative to divorced ones.

**Table 7:** Multivariate regression analysis of hospitalization costs for the without CC/MCC group.

**Explanatory variable**	**Est. (¥)**	**S.E**.	***P-*value**	**95% CI**	**Effect vs. baseline**
Baseline: Divorced + Urban resident basic Med Ins + Traditional surgery	[6,475.43	383.93]	< 0.001	[5,722.95, 7,227.92]	
Age	28.48	6.09	< 0.001	[16.55, 40.42]	+0.44%
Discharge year	342.04	43.56	< 0.001	[256.65, 427.42]	+5.28%
Length of stay	438.00	24.59	< 0.001	[389.81, 486.19]	+6.76%
**Marital status**					
Other	94.75	838.55	0.910	[−1,548.78, 1,738.28]	+1.46%
Widowed	−293.98	264.59	0.267	[−812.56, 224.60]	−4.54%
Unmarried	−233.16	281.37	0.407	[−784.63, 318.31]	−3.60%
Married	−505.30	190.30	0.008	[−878.28, −132.33]	−7.80%
**Medical payment method**					
Urban employee basic Med Ins	1,460.45	270.70	< 0.001	[929.89, 1,991.00]	+22.55%
Poverty assistance	−2,495.01	371.03	< 0.001	[−3,222.21, −1,767.81]	−38.53%
Other	4,592.20	1,158.77	< 0.001	[2,321.04, 6,863.36]	+70.92%
Other social insurance	6,751.22	813.90	< 0.001	[5,155.99, 8,346.45]	+104.26%
Fully government-funded	−289.25	512.35	0.572	[−1,293.44, 714.95]	−4.47%
Fully self-paid	1,270.61	328.04	< 0.001	[627.66, 1,913.57]	+19.62%
Commercial medical Ins	1,084.44	1,311.96	0.408	[−1,486.96, 3,655.84]	+16.75%
New rural cooperative Med	−876.00	279.32	0.002	[−1,423.46, −328.53]	−13.53%
HIFU	3,067.90	399.57	< 0.001	[2,284.75, 3,851.04]	+47.38%
*F*-test	4,536.71		< 0.001		
*R*^2^ Adj.	0.229				

Statistically and economically, large effects of medical payment methods are also evident among patients without CC/MCC. Specifically, compared to the reference category of the Urban Resident Basic Insurance, significantly higher costs are observed under Urban Employee Basic Medical Insurance (increase of 1,460.45 yuan, +22.55%, *P* < 0.001), Other Social Insurance (6,751.22 yuan rise, +104.26%, *P* < 0.001), Fully Self-paid (increase of 1,270.61 yuan, +19.62%, *P* < 0.001), and other insurance (increase of 4,592.20 yuan, +70.92%, *P* < 0.001). By contrast, patients covered by Poverty Assistance (decrease of 2,495.01 yuan, −38.53%, *P* < 0.001) and the New Rural Cooperative Medical Scheme (decrease of 876.00 yuan, −13.53%, *P* = 0.002) incur significantly lower costs. In contrast, patients with fully government-funded and commercial insurance show no significant differences. Finally, the treatment method also induces a major effect: costs for the HIFU procedure are, on average, 3,067.90 yuan higher than those for traditional surgery (+47.38%, *P* < 0.001), underscoring the substantial technical expense associated with HIFU in less complex cases.

**Figure 4:**
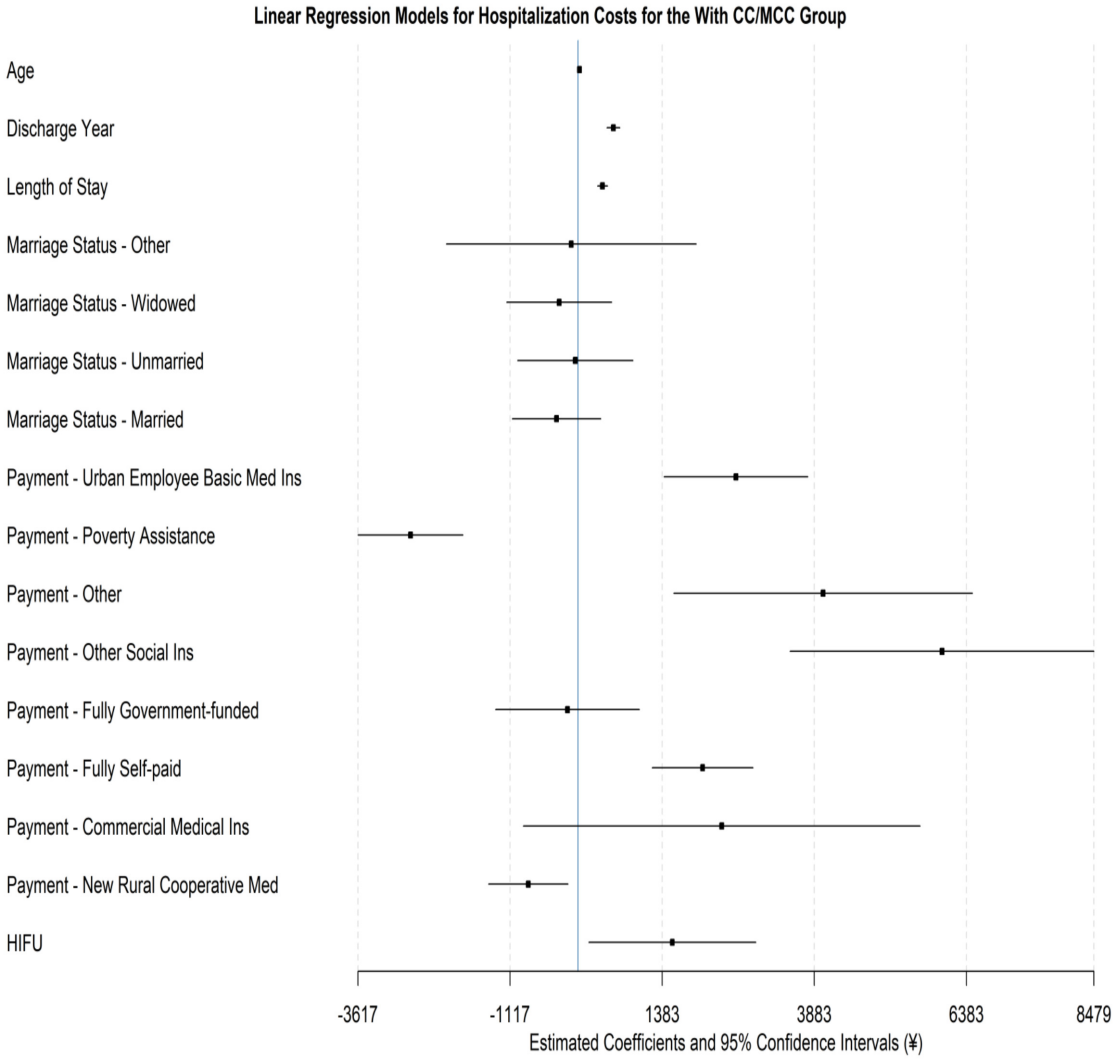
Forest plot for the regression model on hospitalization costs for the with CC/MCC group.

Table 8 and Figure 4 report the regression results for the With CC/MCC group. Using divorced status, Urban Resident Basic Medical Insurance, and traditional surgery as the reference category, the associated average hospitalization cost is 7,183.10 yuan (*P* < 0.001). This is higher than the previously estimated 6,475 yuan for the reference category of Without CC/MCC patients, suggesting an inherently higher cost due to increased clinical complexity. Nevertheless, the relationships between demographic characteristics and hospitalization costs show patterns similar to those documented for patients without CC/MCC. The effects of medical payment methods are also identical, with lower costs for patients under Poverty Assistance (decrease of 2,753.33 yuan, −38.33%, *P* < 0.001) and New Rural Cooperative Medical Scheme (decrease of 818.18 yuan, −11.39%, *P* = 0.014) but significantly higher costs for Urban Employee Basic Medical Insurance, Other Social Insurance, and Fully Self-paid patients.

**Table 8:** Multivariate regression analysis of hospitalization costs for the CC/MCC group.

**Explanatory variable**	**Est. (¥)**	**S.E**.	***P-*value**	**95% CI**	**Change %**
Baseline: Divorced + Urban resident basic Med Ins + Traditional surgery	7,183.10	975.71	< 0.001	[5,270.66, 9,095.54]	
Age	25.09	14.72	0.088	[−3.76, 53.94 ]	+0.35%
Discharge year	581.12	53.87	< 0.001	[475.53, 686.71]	+8.09%
Length of stay	401.33	41.92	< 0.001	[319.15, 483.50]	+5.59%
**Marital status**					
Other	−110.96	1,047.84	0.916	[−2,164.80, 1,942.87]	−1.54%
Widowed	−309.13	440.28	0.483	[−1,172.11, 553.85]	−4.30%
Unmarried	−45.43	484.05	0.925	[−994.19, 903.34]	−0.63%
Married	−353.39	371.56	0.342	[−1,081.67, 374.90]	−4.92%
**Medical payment method**					
Urban employee basic Med Ins	2,595.64	603.31	< 0.001	[1,413.12, 3,778.16]	+36.14%
Poverty assistance	−2,753.33	440.16	< 0.001	[−3,616.07, −1,890.59]	−38.33%
Other	4,027.67	1,252.15	0.001	[1,573.39, 6,481.96]	+56.07%
Other social insurance	5,981.90	1,273.74	< 0.001	[3,485.28, 8,478.51]	+83.28%
Fully government–funded	−174.16	603.26	0.773	[−1,356.58, 1,008.26]	−2.42%
Fully self-paid	2,047.38	422.72	< 0.001	[1,218.83, 2,875.94]	+28.50%
Commercial medical Ins	2,361.86	1,663.14	0.156	[−897.98, 5,621.71]	+32.88%
New rural cooperative Med	−818.18	333.49	0.014	[−1,471.84, −164.51]	−11.39%
HIFU	1,549.52	700.19	0.027	[177.10, 2,921.94]	+21.57%
*F*-test	457.35		< 0.001		
*R*^2^ Adj.	0.227				

More importantly, we observe different impacts of treatment method on costs between the two DRG subgroups. Specifically, while the HIFU procedure raises costs relative to traditional surgery, the magnitude of increase for With CC/MCC patients is much smaller than that for Without CC/MCC patients. For the CC/MCC group, HIFU increases hospitalization costs by 1,549.52 yuan (+21.57%, *P* = 0.027), with a 95% confidence interval ranging from 177.10 to 2,921.94 yuan. This increase is notably smaller than the 3,067.90 yuan estimated for the Without CC/MCC group (+47.38%, *P* < 0.001), with the 95% confidence interval ranging from 2,284.75 to 3,851.04 yuan. From a cost-effective perspective, the benefit of HIFU may be more relevant for clinically complex cases.

## 5 Discussion

This study analyzed DRG subgroup data for uterine fibroid treatments in Sichuan Province (2016–2024), comparing HIFU with traditional surgery and examining key cost drivers. The results confirm that DRG grouping captures meaningful differences in medical resource use and that HIFU demonstrates distinctive cost patterns.

### 5.1 HIFU's enhancement of cost and efficiency

High-intensity focused ultrasound (HIFU), a non-invasive treatment, is increasingly used for uterine fibroids. Using large-sample data, this section shows advantages across four dimensions: first, the cost structure is optimized. In non-CC/MCC cases, consumables were 1.35% with HIFU vs. 10.94% with surgery (*P* < 0.001), and medications were 8.00 vs. 10.39%. In CC/MCC, consumables remained lower with HIFU (2.49 vs. 10.97%), though the gap narrowed. These differences reflect HIFU's non-invasive approach and reduced need for high-value supplies and adjunct drugs. Second, hospitalization efficiency improves. Median LOS was shorter with HIFU (non-CC/MCC: 3 vs. 4 days, *P* < 0.001; CC/MCC: 4 vs. 6 days, *P* < 0.001). In non-CC/MCC, however, higher procedure fees produced a higher median total cost at the index admission despite shorter stays. Third, cost distribution is more stable. Total costs with HIFU showed a centralized pattern, consistent with standardized, operator-independent procedures, whereas surgical costs were more dispersed due to approach choice and intraoperative events, which is useful for budgeting and cost control. Fourth, cost outcomes require nuance. Although consumable and medication shares were lower with HIFU, the treatment-fee share was higher (non-CC/MCC: 50.99 vs. 42.46%), and the median index cost was higher (¥13,590 vs. ¥9,175; *P* < 0.001). Potential downstream advantages (e.g., anesthesia avoidance, faster recovery, uterine preservation) were not measured; our results address index hospitalization only and do not establish overall cost-effectiveness.

### 5.2 Impact of healthcare payment methods on medical resource consumption

Healthcare payment methods are key economic levers shaping clinical decisions and resource use. This analysis reveals several key impacts of healthcare payment methods on medical resource consumption. First, payment type closely correlates with cost levels. Hospitalization costs were higher for UEBMI (+¥1,573.69; +24.41%) and Other Social Insurance (+¥6,700.51; +103.92%), and lower for Poverty Assistance (–¥2,541.92; −39.42%) and NRCMS (–¥873.76; −13.55%; all *P* ≤ 0.002). Drivers likely include: (1) broader coverage enabling costlier treatments; (2) differing enrollee health status and needs; (3) provider responses to insurance status. These gaps highlight the impact of insurance design and the need to strengthen safety nets. Second, DRG systems pose adaptation challenges for HIFU. High fixed costs (equipment, training) require adequate reimbursement, while material savings and efficiency gains can create surpluses. We recommend technology-specific add-on payments to reward innovations like HIFU, paired with strict indication controls to ensure appropriate use.

### 5.3 Impact of DRG subgroup refinement on medical resource consumption

The median hospitalization cost for cases with Complications/Comorbidities (CC/MCC) was significantly higher at ¥14,452 compared to ¥12,476 for non-CC/MCC cases (*P* < 0.001). This gradient manifested through: (1) extended hospitalization by 2 days (9 vs. 7 days); (2) 1.84-percentage-point increase in medication cost share (14.14 vs. 12.30%); (3) 0.39-percentage-point rise in consumables expenditure (10.89 vs. 10.50%). Such stratification demonstrates the sensitivity of DRG subgroups to case complexity, providing a scientific basis for differential payment. Validity was confirmed through a low coefficient of variation (CV ≤ 0.29) and a significant reduction in variance (RIV = 78.27%).

Consequently, hospitals should implement tailored management strategies: optimize workflow efficiency for non-CC/MCC groups while enhancing complication prevention and resource coordination for CC/MCC cases. Simultaneously, payers should dynamically adjust payment standards based on subgroup analyses and establish scientific mechanisms for rate updates.

We acknowledge that excluding abnormal costs could potentially omit a small number of atypical high-cost cases; however, retaining patients with clinically relevant complications in the CC/MCC subgroup reduces the risk of systematic bias in our findings.

### 5.4 Other influencing factors

Beyond treatment modality and healthcare payment methods, patient age, marital status, and length of stay (LOS) were found to significantly influence medical resource consumption. Each additional day of hospitalization increased costs by an average of ¥429.88 (*P* < 0.001). This relationship remained highly significant across both DRG subgroups, indicating that reducing LOS is a critical lever for cost control. Hospitals are advised to enhance efficiency through initiatives like ambulatory surgery management and Enhanced Recovery After Surgery (ERAS) protocols. Costs for older adults patients (aged 55 years and above) increased modestly by 0.44% compared to younger patients (*P* < 0.001), reflecting their higher risk of complications and slower recovery rates. Married patients incurred ¥489.43 lower costs (−7.59%) than divorced patients (*P* = 0.014), likely reflecting the positive role of familial support in rehabilitation. These findings suggest the need for personalized treatment plans and support policies for specific population groups. Costs increased by ¥363.46 (*P* < 0.001) with each additional year in the discharge date cohort, a trend reflecting the combined effects of multiple factors, including advances in medical technology and expanded service provision. This temporal trend must be considered when setting payment standards through implementing scientific rate adjustment mechanisms.

### 5.5 Limitations

Several limitations should be acknowledged. First, the dataset was derived from administrative records. It did not capture clinical details such as fibroid size, symptom severity, or intraoperative complications, which may influence both treatment choice and costs. Second, while outlier costs were excluded to improve subgroup homogeneity, this may introduce selection bias by omitting some atypical high-cost cases. Third, although we adjusted for major demographic and institutional factors, residual confounding cannot be excluded due to the inherent constraints of observational administrative data. Finally, the study was limited to secondary and tertiary hospitals in Sichuan Province; patterns of resource use and reimbursement may differ in other regions of China, which could affect generalizability. These limitations should be considered when interpreting our findings, and future research incorporating clinical indicators and multi-province data would help validate and extend our results.

## 6 Recommendations

In our cohort, length of stay (LOS) varied by treatment and was associated with within-stay charges; HIFU was associated with shorter LOS. Hospitals should optimize clinical pathways and perioperative care to reduce LOS and contain costs. Future work should quantify the marginal cost impact of LOS reductions across DRG strata and treatment types.

### 6.1 Promoting HIFU technology adoption

In uterine fibroid management, HIFU reduced consumables use and shortened LOS, resulting in a more centralized total cost distribution. In non-CC/MCC cases, higher procedure fees led to higher median index costs despite shorter stays. In practice, prioritize HIFU in non-CC/MCC DRG subgroups via clear indication criteria, extending cautiously to selected CC/MCC patients. Standardized operating protocols, specialized training, and patient education can support consistent delivery and acceptance. Longer-term follow-up—capturing recurrence, reintervention, and post-discharge utilization—should clarify the durability of clinical benefits and cost-utility.

### 6.2 Optimizing healthcare insurance payment policies

Under DRG, HIFU's combination of higher procedure fees with material savings and shorter LOS can shift cost profiles and generate potential surpluses. Payment design should incorporate technology-specific add-on payments to offset fixed investments, with differentiated reimbursement tiers aligned to DRG resource consumption. Piloting value-based risk-sharing and surplus-retention mechanisms can reward documented efficiency gains, while tailored reimbursement and supplementary compensation should protect vulnerable groups (e.g., medical assistance recipients, older adults patients). Real-world evaluations should assess the utilization, equity, and budget impact of these adjustments.

### 6.3 Strengthening hospital internal management

HIFU's shorter LOS and more stable cost distribution indicate opportunities to curb unnecessary resource use through standardized care. Hospitals should develop DRG-specific treatment pathways, especially streamlining CC/MCC workflows to reduce redundancy. They should also establish HIFU quality monitoring and routine resource-use audits to ensure safety and efficiency. Additionally, improving coding accuracy for precise DRG classification alongside granular cost-accounting systems is essential for real-time tracking. Studies should examine how pathway standardization and information-system enhancements affect outcomes, DRG assignment accuracy, and cost variance.

This paper analyzes Sichuan DRG data on uterine fibroid treatments, showing that HIFU improves resource use (lower consumables, shorter LOS, and tighter cost dispersion) while, in non-CC/MCC, incurring higher index admission costs due to procedure fees. Coordinated implementation of targeted HIFU deployment, aligned reimbursement, and strengthened hospital management can enhance resource efficiency without compromising quality. Future research should quantify long-term cost-effectiveness, track extended outcomes (recurrence, reintervention, and recovery), and test DRG-aligned cost-containment and payment reforms to guide policy development.

### 6.4 The example of a value-based risk-sharing model for HIFU under the DRG system

A feasible approach is to append an outcomes-based agreement to the existing uterine-fibroid DRG rate for patients receiving HIFU, with settlement over a 12-month horizon tied to clinically meaningful endpoints. Illustrative performance domains include effectiveness (e.g., reintervention rate for repeat HIFU or surgery targeted at ≤ 10% and patient-reported symptom improvement using the UFS-QOL Symptom Severity Score targeted at ≥20-point mean gain), safety (e.g., 30-day procedure-related complications targeted at ≤ 5% and 30-day readmissions targeted at ≤ 3%), and resource use (e.g., LOS of 1–2 days for uncomplicated cases and time to return to regular activity within ≈2 weeks). Payment is adjusted around the DRG base rate: high performance earns a modest quality incentive (for example, a small percentage add-on), near-target performance settles neutral, and underperformance triggers a capped payback (e.g., 10% rebate with a stop-loss limit to avoid excessive downside). To ensure fairness, results are case-mix adjusted (age, fibroid burden, anemia, and prior interventions) and audited via routine claims/EMR linkage or a simple registry. These outcomes and ranges reflect domains commonly reported for HIFU/MRgFUS and are compatible with DRG payment reforms discussed in our references, providing a practical template without changing the core study results.

## Data Availability

The raw data supporting the conclusions of this article will be made available by the authors, without undue reservation.
